# Intake Characteristics as Predictors of Psychotherapy Outcome in a Practice Research Network in Argentina

**DOI:** 10.1007/s10488-024-01394-y

**Published:** 2024-07-12

**Authors:** Javier Fernández-Álvarez, Juan Martín Gómez Penedo, Manuel Meglio, Beatriz Gómez, Anna Babl, Fernando García, Andrés Roussos, Roberto Muiños

**Affiliations:** 1 Polibienestar Research Institute, University of Valencia, Valencia, Spain; 2 Department of Personality, Evaluation, and Psychological Treatments, University of Valencia, Valencia, Spain; 3 Fundación Aiglé, Buenos Aires, Argentina; 4 Asociación Aiglé, Valencia, Spain; 5 Universidad de Buenos Aires, Buenos Aires, Argentina; 6 Consejo Nacional de Investigaciones Científicas y Técnicas, Buenos Aires, Argentina; 7 Adelphi University, New York, United States of America; 8 IPEHCS - CONICET-Universidad Nacional del Comahue, Bariloche, Argentina

**Keywords:** Predictor of Change, Naturalistic Setting, Intake Process, Practice-oriented Research, Practice Based Evidence

## Abstract

There are few studies exploring intake diagnostic characteristics as predictors of change in integrative naturalistic settings. The aim of this study is to explore baseline variables at the intake process and establish the predictive value of the individual trajectories of the patients. We recruited 259 patients undergoing an integrative psychotherapy network of psychotherapists from Buenos Aires, Argentina. Every therapist completed the intake form of each patient involved in the routine outcome monitoring. Thereafter step-wise regressions based on forward selection strategies were used, in order to identify meaningful baseline predictors of patients’ clinical evolution, derived from the intake process. The selected predictors were social support network, subjective distress, the initial measure of clinical distress, unemployment, sociocultural status and reactance. When including those six variables in a multilevel model, the results indicate that social support network, subjective distress, and the initial measure of clinical distress were significant predictors of the trajectories of OQ-30, whereas unemployment, sociocultural status and reactance were not significant. The results regarding social support network are in line with the literature, while results of socioeconomic status (unemployment and sociocultural level) move in an opposite direction in comparison to the available evidence. Moreover, the mental health findings (initial OQ-30 and subjective distress) confirm the contradictory body of literature produced in this domain. Finally, reactance seems to be a significant predictor in previous study in contradiction of our results. Overall, this endeavor constitutes important but preliminary evidence to enhance the production of bottom-up science within practice research networks in the global south.

In the realm of psychotherapy, the quest for personalization has long been a central focus (Paul, 1967). While this interest has persisted for many years, its importance has surged in recent times (Cohen et al., 2021; Delgadillo & Lutz, 2020; Zilcha-Mano, 2021). One significant consequence of the limited development in this area is the enduring gap between practitioners and scientists. This gap is rooted in the dichotomy between research, which often emphasizes standardization, and psychotherapy practice, which focuses on individual needs. Shifting towards an understanding of what works for each individual, based on their clinical needs and preferences, promises substantial progress in integrating clinical practice with research (Lutz et al., 2021). In recent years, there have been notable advancements in the precision of measurement methods (Lutz et al., 2022). These methodological improvements have facilitated a better understanding of how change occurs in psychotherapy and for whom it is effective (Zilcha-Mano, 2021; Zilcha-Mano & Webb, 2022).

The most comprehensive existing framework for personalized interventions organizes them along three dimensions: time (when the intervention is delivered), level (the intensity of the intervention), and structure (method of personalization from artisanal to statistical models). In terms of the timing, interventions can be tailored before treatment begins, during treatment, or after treatment has concluded (Cohen et al., 2021). Assessing patients at baseline and tailoring intervention strategies to their specific needs and preferences offers significant potential for treatment personalization. Traditional diagnostic frameworks often fail to capture the nuanced presentations common in clinical practice, as patients frequently exhibit complex symptomatology, comorbidities, and subthreshold conditions (Howard et al., 1996). Therefore, relying solely on diagnostic labels to inform treatment strategies is insufficient. A multitude of patient-specific factors and contextual variables, such as symptom severity and sociodemographic characteristics, are crucial determinants of treatment outcomes (Lutz et al., 2021). The interaction between individual patient characteristics and therapeutic trajectories is essential for improving case assignment.

Identifying predictive and prescriptive variables at baseline can lead to models that recommend the most suitable treatment approaches or strategies for specific individuals at the appropriate time. This traditional approach to treatment personalization involves distinguishing between predictive variables, which indicate a general association with outcomes, and prescriptive variables, which identify subgroups that respond differently to various treatments (Lutz et al., 2021).

Two main approaches have been used in the literature to identify predictors and moderators: theory-driven and data-driven (Zilcha-Mano, 2019). Theory-driven, or “top-down,” approaches are based on theoretical conceptualizations about which subpopulations benefit most from specific treatments. Decades of research have produced substantial evidence for various sociodemographic and clinical variables (Constantino et al., 2021). The most pioneering effort in this regard is systematic treatment selection, which goes beyond symptom-based diagnoses to consider a broader array of clinical and sociodemographic factors in treatment planning (Beutler et al., 2016). Conversely, data-driven, or “bottom-up,” approaches often employ machine learning techniques to identify moderators from a wide range of potential variables, some of which may be chosen based on relevant prior theories (Cohen & DeRubeis, 2018). Recently, there has been a shift towards data-driven methodologies (Cohen et al., 2021; Delgadillo & Lutz, 2020). However, both strategies are crucial for advancing psychotherapy.

Lutz and colleagues (2021) provide an overview of predictors contributing to therapeutic outcomes. Although the field has not yet made significant progress enough to provide replicated evidence regarding the prognostic markers, a growing body of literature has been produced that has built a more solid understanding of these predictors. In turn, Lutz et al. (2021) organized the predictors into four categories, which are patient factors, therapist factors, therapy factors and contextual factors.

Despite these advances, much existing research is based on controlled environments, potentially limiting its applicability to real-world clinical settings (Castonguay et al., 2013). Another limitation is the lack of studies conducted in underserved countries. In low- and middle-income countries (LMICs), nearly 80% of people with mental disorders live (WHO, 2022). Paradoxically, 90% of the research has been conducted not only in non-LMICs but also in certain privileged populations within those countries (Fonagy & Luyten, 2021).

However, there are notable exceptions. For instance, a practice-based research infrastructure in Kenya has conducted significant studies on psychotherapy effectiveness and service quality in public hospitals (Falkenström et al., 2017). One study by Kumar et al. (2018) found that younger patients showed more improvement than older ones throughout treatment, and patients seen by interns experienced higher levels of distress and greater improvement than those seen by professionals. Within the same sample, Falkenström et al. (2019) examined the predictive value of the therapeutic alliance, finding it to be a robust predictor of distress, consistent with evidence from the Global North (Zilcha-Mano & Fisher, 2022).

In Latin America, relevant research includes a study by Zilcha-Mano and Errázuriz (2015) in Chile, which highlighted significant associations between alliance trajectories and outcomes, influenced by factors such as symptom severity and treatment duration. Within the same clinical sample, Behn et al., (2018) found that the relationship between symptom distress and life satisfaction change varies according to the family income of the patients. For high-income patients, an increase in symptom distress predicts an increase in life satisfaction. but not the reverse. Conversely, for low-income patients, an increase in life satisfaction predicts a decrease in symptom distress, but not vice versa.

In Argentina, Gómez Penedo et al. (2019) explored the relationship between alliance and outcome in patients with emotional disorders, finding that patients with overly nurturant styles showed a stronger relationship between alliance negotiation and early treatment progress. Another Argentinian study on emotion regulation and outcomes in an integrative clinical and training center demonstrated that both between-patient and within-patient effects were related to improvements in emotion regulation (Fernández-Alvarez et al., in press).

Our study investigates the predictive value of intake characteristics on outcomes in integrative psychotherapy within a naturalistic context. Analyzing these variables within a practice research network aims to illuminate individual trajectories of change and enhance treatment personalization by anticipating the specific needs of patients. Specifically, we examine pretreatment variables within an integrative psychotherapy framework in Buenos Aires, Argentina, to elucidate their predictive value for individual treatment trajectories.

## Methods

### Participants

The sample consisted of 259 patients who were treated in a psychotherapeutic outpatient clinical and training center in Buenos Aires, Argentina. To be eligible, patients had to be at least 18 years old and have a main diagnosis based on the *Diagnostic and Statistical Manual of Mental Disorders* (5th ed.; *DSM–5;* American Psychiatric Association [APA], 2013). The exclusion criteria were (a) severe personality disorder, (b) acute suicidality, (c) substance abuse, or (d) assignment to couple therapy, group therapy, or family therapy within or outside the center.

On average, patients were 32.25 years of age (*SD* 12.37). Most participants were female (61.48%) and had at least completed or were enrolled in a professional training program or a university program (68.33%). Information about ethnicity was not collected. The distribution of principal disorders in the sample was as follows: 17.76%, depressive disorders; 2.31% adaptative disorder; 21.23% anxiety disorders; 6.95%, obsessive–compulsive disorder; 2.70% posttraumatic stress related disorders; 6.56%, personality disorders; 2.70% bipolar disorder; 1.93 somatoform disorder; 22.01% others; and 15.83% were missing. Before the intake interview, patients gave their written informed consent to use the intake assessment and the routine outcome monitoring for research purposes.

### Therapists

Seventy therapists participated in the study. The caseloads ranged from 1 to 19 patients per therapist (*median* 2; *SD* = 3.76). All therapists were Argentinians, but no information about ethnicity was collected. As part of their postgraduate training, all the therapists received the same 2-year training course in integrative cognitive behavioral therapy. Furthermore, all the therapists had biweekly video-based supervision meetings in small groups of between 6 and 8 people, conducted by experts in the respective diagnoses.

### Treatment

The psychotherapy model implemented in the clinical and training center has a cognitive behavioral core but incorporates concepts and procedures from psychodynamic, systemic, and humanistic-existential therapies (Fernández-Alvarez, 2001). It is therefore based on (1) a broad information processing framework utilizing an integrated conceptualization of theory of mind (Osbeck, 2009); (2) a biopsychosocial psychopathological model with personality as the core structure that organizes the experience and behaviors and accordingly their dysfunctions (Livesley, 2012); and (3) principles of change that guide the respective interventions throughout the treatments (Castonguay & Beutler, 2006). Hence, treatments are not manualized, but they rather follow these principles of change as well as phases of treatment.

### Materials

**Outcome Questionnaire (OQ-30.2)** - Outcome was assessed at baseline, then the five first weeks and then every four sessions using the Outcome Questionnaire (OQ-30.2; Ellsworth et al., 2006). The OQ-30.2 consists of 30 items ranging from 0 (never) to 4 (almost always), totaling a maximum score of 120 points. This scale evaluates three independent dimensions: symptomatology, interpersonal relationship and overall functioning and additionally a total score. The OQ-30.2 has been found to have good internal consistency (alpha 0.90), good concurrent validity (with the Depression Anxiety Stress Scales: *r* = 82) and sensitivity to change. The Spanish version of the OQ 30.2 (Errázuriz et al., 2017) presents good psychometric properties. In the current sample the internal consistency was excellent (Cronbach’s α = 0.91).

**Initial interview -** At the beginning of each therapeutic treatment, patients are contacted by the therapist to conduct the initial interview, followed by the baseline assessment of self-report measures and the intake form. The detailed description of the baseline assessment of self-report measures is beyond the scope of this article but it includes a core battery of measures (usually interpersonal problems, symptomatology, pathological personality) and an individualized set of measures tailored to each patient’s specific problems (refer to Fernández-Alvarez et al., 2015; 2022for a more comprehensive description).

**The Intake form** is completed by the therapists based on the initial interview. It consists of numerous variables that include sociodemographic and clinical information. A selection of 22 variables was made based on the following criteria: (1) clinical relevance; (2) previous evidence as relevant predictors in psychotherapy; and (3) data availability across the entire sample of patients regardless of their condition and treatment modality. Some variables were excluded for being too sensitive (e.g., personal address) or not feasible to include in the models, such as the genogram.

From the selected 22 variables, 23 predictors are processed given that one variable (locus of control) is decomposed into two different predictors. The final selection of predictors were: gender identity (male cisgender/female cisgender), civil status (in a relationship/others), unemployment (yes/no), educational level (high/low), cultural level on a likert scale of 1 to 3 (low, mid, high), socioeconomic level on a likert scale on 1 to 5 (low, middle low, mid, middle high, high), duration of distress (equal or less than 2 years or more than 2 years), previous psychotherapeutic consultation (yes/no), same reason of previous consultation (yes/no), number of previous psychotherapeutic treatments (from 0 to 4 which is more than 3), number of previous psychopharmacological treatments (from 0 to 4 which is more than 3), the stability of patient’s attributional hypothesis (yes/no), internal locus of control (yes/no), oscillation of locus of control (yes/no), wide social support network (yes/no), level of clinical severity on a likert scale of 0 to 4 (“non-clinical”, “mild”, “moderate”, “severe”, “extremely severe”). Treatment expectations and reactance were assessed on a likert scale 1 to 5 (“low” to “high”). Perception of social support and perception of family support were assessed on a likert scale of 1 to 3 (“low” to “high”). Change motivation on a likert scale of 1 to 5 (“none” to “high”), subjective distress on a likert scale of 1 to 5 (“mild” to “very intense”).

The first step was to test the reliability of the intake form as a clinical instrument. With that aim, a consistency analysis was carried out through an inter-rater study to determine the degree of homogeneity in the completion of the instrument by the therapists in charge of the intake process. To conduct the consistency analysis, five intake interviews conducted by the same interviewer were selected. These interviews were recorded on video. Four expert psychologists specializing in the intake process of patients were selected to serve as external judges. Each of the four external judges individually attended and completed the intake form for each of the five recorded intake interviews. It is relevant to note that all four external judges evaluated the same five interviews. To facilitate the inter-rater study, training was designed, providing user instructions to the same four External Judges who participated in the previous analysis. The information from the completed Intake Forms was assessed, and the Fleiss’ Kappa coefficient was calculated to assess the level of interrater reliability agreement.

### Procedure

This study took place in an integrative cognitive-behavioral therapy clinical and training center between December 2019 and March 2023. Of the 750 patients received during this period, those who met the eligibility criteria and completed the Outcome Questionnaire 30 (OQ-30) were included in the sample. The patients are part of two circuits. In the first circuit, they are referred from various sources, such as other patients, professionals, current or former students, or other organizations and pay a basic institutional fee. In the second circuit, they are part of the Therapeutic Care Program for People with Limited Resources program and are referred to by other community organizations. Therapeutic Care Program for People with Limited Resources is the acronym for PATER and consists of a sliding scale fee program. The therapists involved in the study, who are part of the clinical center, dedicate a significant number of hours to patient care. Additionally, these therapists voluntarily participate in the scientific development area and the research process chain. The internal functioning is structured according to a rotating role model among its members, covering professional, academic, management, administrative tasks, among others. A practice-research network was implemented 35 years ago at this clinical and training center. Following Practice Oriented Research principles (Castonguay et al., 2013), clinicians were involved in the design and setup of the project.

The therapists involved in the intake process completed the intake form after the first interview with the newly admitted patients. Subsequently, these patients were part of the routine outcome monitoring implemented for patients at the clinical center. The monitoring consisted of answering the Outcome Questionnaire 30 (OQ-30) the first five sessions, then every two sessions until session fifteen and then every four sessions. The therapists did not receive any feedback concerning patients’ ratings on the OQ-30.

### Analytic Strategy

To assess the level of inter-rater agreement on the intake form, the Fleiss’ Kappa coefficient was calculated. Fleiss’ Kappa is a statistical measure that assesses the reliability of agreement among a fixed number of judges who assign categorical or ordinal scores to a set of items. Unlike Cohen’s Kappa, this indicator allows for the use of more than two judges. Fleiss’ Kappa measures the degree of agreement beyond what would be expected by chance (Fleiss et al., 2003). To assess the magnitude of the obtained agreement, we can refer to the table presented by Landis and Koch (1977), which indicates slight agreement for Kappa values between 0.2 and 0.4 and moderate agreement for values between 0.4 and 0.6.

To narrow down predictors from the myriad of variables available in the intake form, we initially fitted linear and quadratic multilevel models incorporating different time variables as single predictors, along with fixed and random effects to predict the outcome variable. The linear model with random effects provided the best fit, from which we extracted empirical Bayes estimates of slope as individual estimations of the time effects, representing individual trajectories. Additionally, we conducted a forward regression to predict individual slopes. This is a stepwise regression that seeks to fit models in which the choice of predictors is carried out by an automatic procedure.

Having selected the six predictors indicated as most important by our forward regression analysis, we used hierarchical linear models (HLMs; Raudenbush & Bryk, 2002). HLMs address the dependency of data due to repeated measures, providing a robust strategy to handle missing data within patients. These models mimic an intent-to-treat approach, allowing for the inclusion of all participants with at least one measurement point in a given outcome variable into the analyses (see Westra et al., 2016). Before entering predictors into the model, we mean-centered them. Categorical variables were dichotomized, with dichotomized variables centered as 0 and 1, while ordinal variables were treated as quantitative variables.

The study was approved by the University of Buenos Aires. All analyses were conducted using the free software environment *R* software version 2023.06.2.0 + 561 (R Core Team, 2023). To measure interrater reliability, we computed Fleiss’ κ using the kappam.fleiss function of the irr package. To run the multilevel analyses, the package lme4 version 1.1.21 was used (Bates et al., 2015). To illustrate the results of the study, we created figures with the package ggeffects version 0.14.0 (Lüdecke, 2018). As an effect size measure for significant effects of the multilevel models, we computed standardized coefficients.

## Results

### Interrater Reliability

To assess individual differences between clinicians, a subgroup of five patients was evaluated by the same four clinicians, facilitating specific measures of interrater reliability. For this sub population, the weighted Kappa scores were 0.56, indicating moderate agreement (Landis & Koch, 1977).

### Sample Descriptives

In Tables 1 and 2, we present the descriptive statistics of the main variables of the study at baseline and the estimated session-by-session rates of change of the outcome variable with repeated measures.

**Table 1:** Sample descriptives of numeric variables

Variables	Sample descriptives at baseline		
	Mean	*SD*	Theoretical range
Initial clinical distress (OQ-30)	45.11	16.58	[0, 4]
Age	32.25	12.38	
Subjective distress	2.49	0.89	[0, 4]
Past psychotherapy	0.76	0.43	[0, 4]
Psychopharmacology	0.35	0.48	[0, 4]
Attribution Control	1.63	0.75	[1, 4]
Representation intake team	1.77	0.75	[0, 4]
Expectations to change	3.33	0.85	[1, 5]
Motivation to change	3.45	0.85	[1, 5]
Reactance	2.39	0.99	[1, 5]
Social Support Received	1.82	0.64	[1, 3]
Family Support Received	1.99	0.72	[1, 3]

**Table 2:** Sample descriptives of categorical variables

Gender	
Female cisgender	61.5
Male cisgender	38.5
*Attributional hypothesis*	
Stable	68.9
Unstable	28.0
Neutral	3.1
*Locus of control*	
External	14.0
Internal	61.1
Neutral	1.2
Oscillating	23.7
*Social support network*	
Wide	33.5
Narrow	66.5
*Previous consultation*	
Yes	76.0
No	24.0
*Reason of previous consultation*	
Similar to the current reason	43.7
Different to the current reason	35.4
Both similar and different	20.8
*Distress duration*	
Up to 10 years	7.8
Up to 2 years	36.6
Up to 3 months	25.7
Up to 6 years	12.5
More than 10 years	17.5
*Socioeconomic status*	
High	0.4
Middle-high	23.6
Middle	54.1
Middle-low	10.9
Low	11.2
*Sociocultural status*	
High	13.9
Middle	77.6
Low	8.5
*Educational level*	
Incomplete high school or below	3.9
High School	12.0
University in progress	38.2
University completed	27.0
Posgraduate in progres	14.3
Posgraduate completed	4.6
*Employment*	
Employed	80.6
Unemployed	9.0
Others	10.4
*Civil status*	
Married	17.8
Separated	5.4
Single	61.4
Widow	1.54
Coliving	14.3

### Primary Analyses

The fully unconditional model showed that patients presented an estimated score of 39.93 in OQ-30.2 across treatment. The model indicated that the patient level explained 78% of variance in the outcome variable (ICC = 0.78).

*The unconditional time-as-only predictor model* showed that linear time was the best fit. Patients tended to reduce their OQ-30.2 scores by 1.38 units per measure, γ_10_ = -1.38, *SE* = 0.16, 95% CI [-1.70, -1.06], *t*(88) = -8.54, *p* < 0.001. This means that there was a significant reduction of 1.38 in OQ-30.2 levels over the course of the treatment measure by measure.

The selected predictors of the forward regression indicated that unemployment (β = -0. 29; 95% CI [-0.65, -0.72]), wide social support network (β = -0. 19; 95% CI [-0.43, 0.03]), reactance (β = 0.05; 95% CI [-0.05, 0.16]), sociocultural status (β = 0.14; 95% CI [-0.09, 0.36]), initial measure of OQ-30.2 (β = -0.03; 95% CI [-0.04, -0.02]), and subjective distress predicted (β = 0.14; 95% CI [0.02, 0.26]) the individual trajectories of change. In Table 3 the results are presented.

**Table 3:** Summary of regression analysis by forward selection

Variables	β	t	*R*	*R*²	∆*R*²	AIC	95% CI
Model			0.53	0.28	0.01	648.8	
Initial measure of OQ-30	-0.03	-8.882***					[− 0.037, − 0.024]
Subjective distress	0.14	2.220*					[0.015, 0.258]
Social support network	-0.20	-1.710					[− 0.431, 0.030]
Unemployment	-0.29	-1.573					[− 0.647, 0.072]
Sociocultural status	0.14	1.189					[− 0.090, 0.364]
Reactance	0.05	1.024					[− 0.051, 0.160]

Note: *N* = 259; *p < 0.05, **p < 0.01, ***p < 0.001

We then included these predictors in a multilevel model. Including time as a random effect significantly improved the model fit compared to a model including time as a fixed effect, χ^2^(2) = 272, *p* < 0.001. This model revealed significant effects for wide social support network, subjective distress, and initial measure of OQ-30.2, while unemployment, sociocultural status and reactance were nonsignificant. In Table 4 the results are presented.

**Table 4:** Main effects models

	OQ-30		95% CI	
Fixed Model Effects	γ	*SE*	Lower	Upper
***Main effects***				
Intercept	42.04*	0.46	41.13	42.95
*Wide Social support network*	-0.75*	0.37	-1.88	-0.46
*Subjective distress*	0.45*	0.20	0.06	0.84
*Initial clinical distress OQ-30*	-0.07*	0.01	-0.09	-0.05
*Unemployment*	-0.71	0.60	-1.88	-0.46
*Sociocultural level*	0.38	0.40	-0.41	1.18
*Reactance*	0.14	0.17	-0.20	0.48
*Model comparison*	χ^2^(2) = 272.48, *p* < 0.001			

OQ-30: Outcome Questionnaire 30; *p < 0.001

**Fig. 1:**
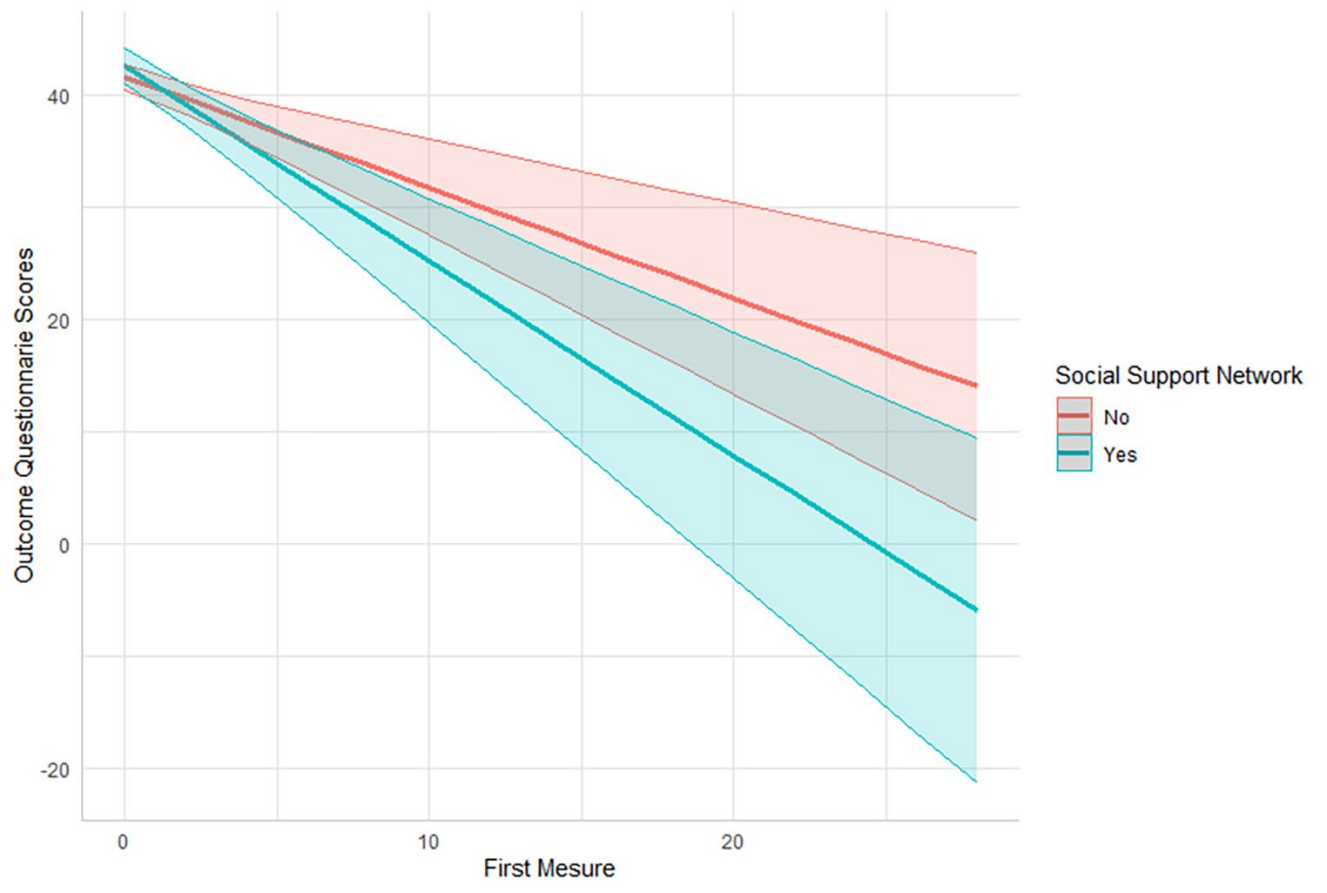
Social support network as a predictor of patient’s trajectories

**Fig. 2:**
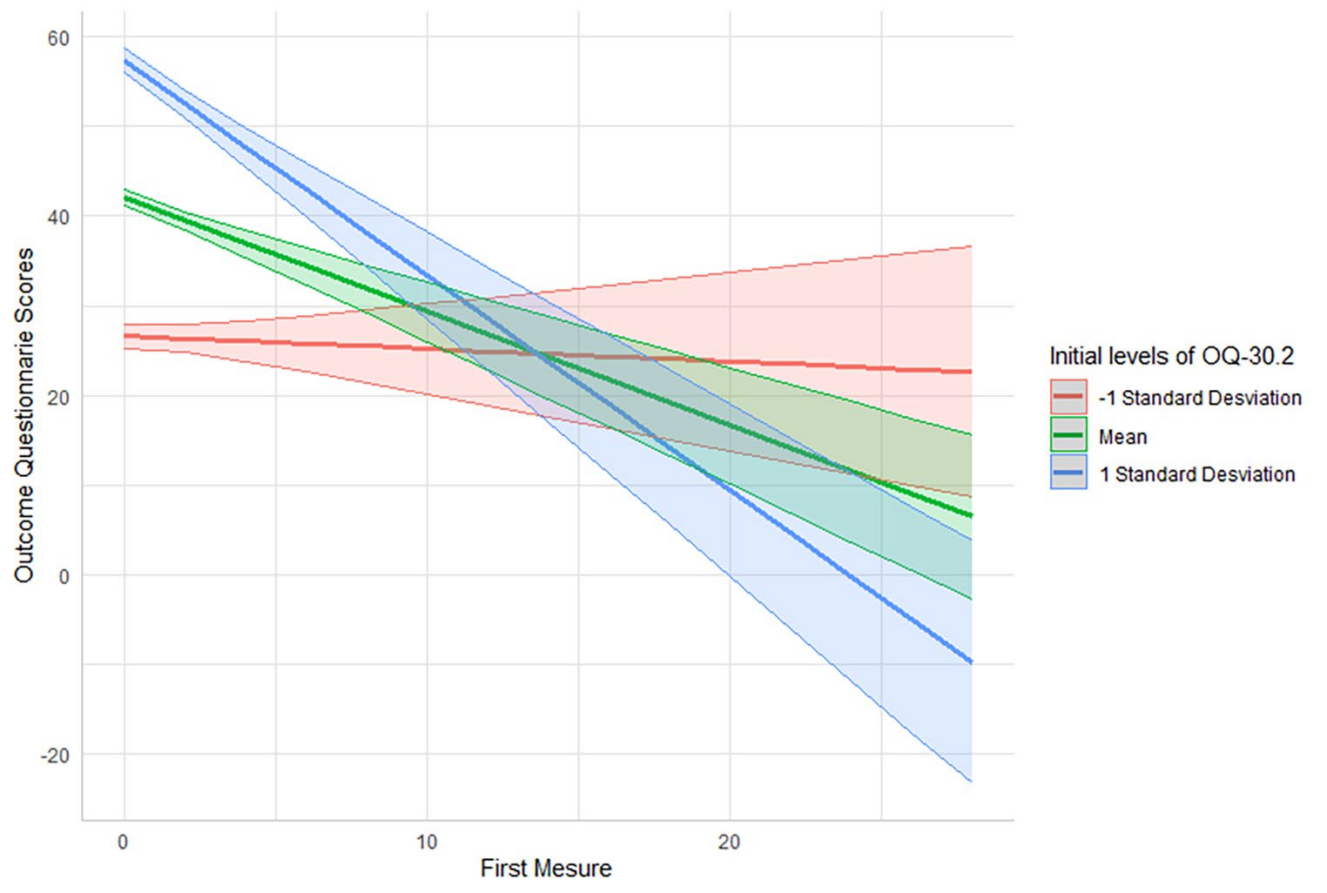
Initial levels of OQ-30.2 as a predictor of patient’s trajectories

**Fig. 3:**
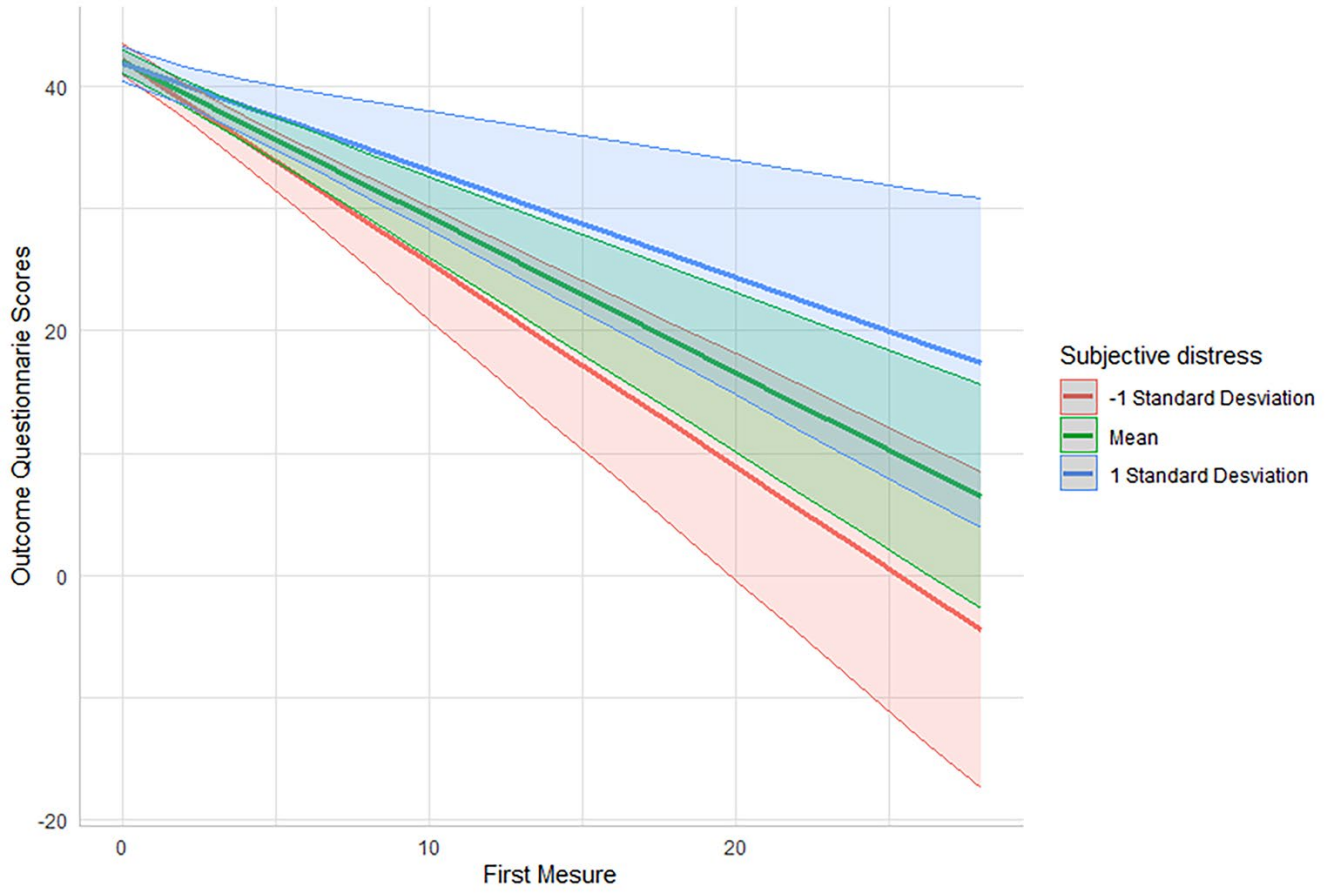
Subjective distress as a predictor of patients’s trajectories

The significant effect for wide social support network, γ_01_ = -0.75, *SE* = 0.37, CI 95% [-1.88, -0.46], *t*(79) = -2.04, *p* = 0.045, meaning that individuals with a wide social support network presented a significant decrease of 0.75 units in comparison to the people without a wide social support network on the OQ-30 on a measure-by-measure basis. In the case of subjective distress, γ_02_ = 0.45, *SE* = 0.20, CI 95% [0.06, 0.84], *t*(91), *p* = 0.026, those who had lower subjective distress presented a significant decrease of 0.45 units on the OQ-30 measure by measure. Besides, those with higher initial levels of clinical distress γ_03_ = 0.07, *SE* = 0.01, CI 95% [-0.09, -0.05], *t*(91), *p* < 0.001, presented a significant decrease of 0.07 units on the OQ-30 measure by measure. Regarding the effect for unemployment, γ_04_ = -0.71, *SE* = 0.60, CI 95% [-1.88, 0.46], *t*(111) = -1.19, *p* = 0.24, sociocultural status, γ_05_ = 0.37, *SE* = 0.40, 95% CI [-0.41, 1.18], *t*(86) = 0.92, *p* = 0.362, and reactance, γ_06_ = 0.14, *SE* = 0.17, 95% CI [-0.20, 0.48], *t*(87) = 0.83, *p* = 0.41, were not significant. Figures 1, 2 and 3 illustrate the significant results.

## Discussion

The aim of this paper was to analyze pretreatment variables using the intake form of Aiglé Foundation and assess its predictive value in relation to the individual trajectories of patients undergoing integrative psychotherapy. First, we assessed the reliability of the intake form to explore its predictive nature in the naturalistic setting where it was initially implemented.

Our analysis identified six significant predictors: unemployment, sociocultural status, subjective distress, clinical distress at the first measure of the routine outcome monitoring, reactance, and wide social support network. These six predictors were included in a multilevel model, which demonstrated that wide social support network, subjective distress, and initial levels of clinical distress were statistically significant predictors of the patients’ trajectories. The predictors in our final model can be categorized into three of the four groups presented by Lutz et al. (2021): wider context, therapy processes, and patient factors.

### Wider Context

Having a wide social support network emerged as a significant predictor, aligning with existing evidence that social support is associated with improved psychotherapy outcomes. Constantino and colleagues (2021) highlight that social support has a small but significant effect on treatment outcomes, irrespective of diagnosis and theoretical approach. Previous research indicates that social support is linked to better outcomes in long-term therapy (Lindfors et al., 2014) and a higher likelihood of returning for additional therapy (Kilcullen et al., 2021). This suggests the importance of considering social support network at the beginning of treatment to anticipate potentially difficult trajectories of change.

Furthermore, unemployment and sociocultural level can be interpreted as proxies for socioeconomic status, which encompasses various factors reflecting an individual’s position within society and their access to resources, including income, personal and family wealth, occupation, and education level. Contrary to prevailing literature, which often suggests that lower socioeconomic status correlates with less improvement in therapy, our findings did not show this association. A meta-analysis using employment status as a proxy for socioeconomic status found a modest but significant link between unemployment and reduced improvement (Finegan et al., 2018).

One possible explanation for the lack of significance within this sample, contrary to existing evidence, may be attributed to the presence of the PATER program. This initiative involves dedicating time and resources within the organization to tailor interventions for patients from low-income backgrounds (Fernández Alvarez et al., 2022). While not all participants in our study were enrolled in this program, it is noteworthy that all therapists underwent training in its principles.

These principles include fostering adherence and motivation among patients who might lack prior exposure to psychotherapy. Notably, previous psychotherapy experience could be a confounding factor, given the extensive psychotherapy tradition in Argentina. Individuals who are part of PATER navigate various filters with social organizations before starting a psychotherapeutic process, and addressing their expectations is pivotal in these cases. The available literature emphasizes the importance of therapists recognizing the diverse components of their patients’ socioeconomic status and acknowledging their potential to act as facilitative or risk factors for treatment outcomes (Constantino et al., 2021), which is pivotal for the training and therapeutic work of the therapists at this clinical program (Fernández-Alvarez et al., 2022).

### Patient Factors

Both subjective distress and initial levels of OQ-30 scores can be considered as patient factors, concretely as mental health predictors (Castonguay et al., 2021). Our findings indicate a negative relationship between subjective distress at intake and therapeutic outcomes. Specifically, patients reporting higher subjective distress at intake tended to show less improvements in their trajectories of clinical distress. Conversely, higher initial scores of the OQ-30.2 were associated to more improvements in their trajectories of clinical distress. Although these might be interpreted as contradictory results, there are potential explanations for these findings.

The understanding of how symptom severity predicts treatment outcomes remains complex and contested within the literature. While some past studies have suggested a link between higher initial symptom severity and unfavorable treatment results, others have unveiled a contrasting pattern (Constantino et al., 2021). For instance, a study focused on patients with borderline personality disorder found that though higher initial severity was linked with poorer outcomes, symptom severity did not significantly correlate with therapeutic effectiveness (Kvarstein et al., 2019).

As noted by Constantino et al. (2021), there are likely interacting variables moderating this effect, such as the treatment type, clinical setting, or the specified clinical condition being addressed. Furthermore, how severity is defined and measured is of paramount importance. While the link between symptom severity and impairment chronicity tends to yield more consistent results, the association with general symptom severity is less straightforward. Notably, when assessing problem severity through a multidimensional lens, greater problem-related distress can be positively related to therapeutic improvement in cases without a concurrent risk of suicide or homicide (Uckelstam et al., 2019).

It is important to highlight that both variables -subjective distress and initial scores of clinical distress- were included as covariates within the same model. This implies that the effect of each variable was estimated when accounting for the influence of the other. Results suggest that the contributions of self-reported clinical severity, derived from patients’ perceptions of their symptoms, interpersonal relationships, and social functioning, were distinct from the effects of patients’ overall distress perception on outcome prediction. The complexity of measuring severity at the beginning of the treatment is evident, and depending on the operationalization and instruments used, results can meaningfully vary. Previous findings suggest that predictions about individual treatment progress might be more accurate for specific domains of psychological impairment than for general distress (Mütze et al., 2022).

### Therapy Processes

The only selected predictor included in our final model that pertains to the category therapy processes was reactance, which refers to an oppositional tendency to avoid making the changes recommended by the therapist due to apprehension or an aversion to change (Beutler et al., 2018). While ample evidence shows a significant connection between reactance and patients’ characteristics, there is no conclusive direct association between reactance and outcome. Instead, a meta-analysis comprising 1,208 patients shows that patients high in reactance tend to have better outcomes when therapists are less directive, whereas patients lower in reactance tend to have better outcomes when therapists are more directive (Beutler et al., 2018). Given that the present study did measure therapists’ directiveness, caution is needed when interpreting these results considering the available evidence.

### Clinical Implications

These results can constitute a valuable contribution to various aspects of the clinical process. They allow for informed decision-making in the final phase of the patient intake process when formulating the treatment plan. With these kinds of results, it can be predicted that certain patients will be at risk of poorer outcomes. This data provides information to the therapist about the possible trajectory, enabling them to address obstacles early on, strengthen treatment adherence, and prevent dropouts. Clinical decisions can be made accordingly to increase the likelihood of success, such as increasing monitoring throughout the treatment, providing more thorough supervision for the patient, among other strategies. Furthermore, in the area of therapist training, these results help identify the need for developing competencies in strategies that address the influence of these predictors. Especially, multicultural competencies are of key importance given the context in which this practice research network is developed.

### Limitations

The results, while valuable for the novelty of the context in which study was conducted, must be analyzed in light of several limitations. Firstly, despite efforts to improve the reliability of the intake form, the results suggest that the instrument does not provide strong interrater reliability. Consequently, these results cannot be generalized and must be considered as an important exploratory endeavor that needs to be replicated. Moreover, it is worth highlighting that the preferred approach would involve assessing the outcome measure (the OQ-30) prior to each therapy session. However, due to the structure of the clinical and training center, this ideal procedure was not feasible, and instead, both measurements were conducted post-session. Lastly, it must be considered that this is a naturalistic study embedded in a practice research network and although it constitutes a strength in terms of the articulation of research and practice, there is considerable missing data which although multilevel models allow for the estimation of the trajectories even in the event of missingness but it undeniable that it is a source of potential bias.

### Future Lines

In moving forward, several avenues for further research merit consideration. First and foremost, while predictors indicating patient variables that are associated with outcomes provide important clinical implications, they can be more informative when exploring their differential role in certain subpopulations, contexts or therapists. This is particularly true when predictors have a prescriptive or moderating character (Lutz et al., 2021). For example, incorporating information about therapists can help identify patient profiles that may benefit from certain therapist characteristics, in line with Beutler et al. (2016), and thereby enable evidence-based decisions for selecting therapists.

In a similar vein, exploring the trajectories of change for different mechanisms and outcome variables would help disentangle specific aspects that may be important for certain groups of patients. For example, in Latin America, evidence shows that patients with lower family incomes benefit more from improvements in life satisfaction, which predict reductions in clinical distress. However, these results are not found in high-income patients (Behn et al., 2018).

With an increased sample size, it would be also feasible the development of machine learning algorithms that can offer concrete clinical tools to support the intake process for incoming patients. This expansion can extend beyond merely pre-treatment variables and encompass the whole treatment process, taking into account treatment strategies and procedures. In conjunction with other standardized intake assessments, these machine learning algorithms hold promise for predicting patient outcomes within the clinical center. Such an endeavor represents a crucial step towards the development of computer-assisted feedback systems, akin to existing examples in Northern Europe and North America (Lutz et al., 2021).

Furthermore, beyond the examination of treatment trajectories, a critical avenue of research involves delving into the mechanisms of change, elucidating not only for whom interventions are effective but also how they operate (Gómez Penedo et al., 2022; Moggia et al., 2023). By integrating both research paradigms—understanding how psychotherapy functions and for whom—through the examination of the interplay between predictive and prescriptive variables with mediators of change, we can advance the development of nuanced machine learning algorithms aimed at personalizing treatments and intervention strategies in precise and tailored ways.

However, to achieve these goals, it is imperative to foster the implementation process of the practice research network. Implementation science and practice-oriented research have an evident convergence and complementarity (Youn et al., 2023) that need to be harnessed to improve the recruitment of data for clinical purposes and relevant research. In this sense, monitoring therapeutic processes is essential, as is implementing the intake form and the initial self-report assessment at the beginning of treatments to recruit significant data. This data enables the creation of an ever-growing practice-oriented research infrastructure where clinicians use research outputs to improve their clinical resources (e.g., clinical support tools derived from computer-assisted feedback systems). Our collaborative efforts seek to bridge the gap between theoretical insights and practical application, ultimately optimizing treatment outcomes for individuals undergoing integrative psychotherapy.

In conclusion, the present study furnishes exploratory findings that serve as a foundational steppingstone for the elaboration of bottom-up knowledge within the context of a practice research network situated in the Global South. These findings illuminate potential directions for future research endeavors aimed at enhancing the efficacy and personalization of psychotherapeutic treatments.
